# WAO Guideline for the Management of Hereditary Angioedema

**DOI:** 10.1097/WOX.0b013e318279affa

**Published:** 2012-12-15

**Authors:** Timothy Craig, Emel Aygören Pürsün, Konrad Bork, Tom Bowen, Henrik Boysen, Henriette Farkas, Anete Grumach, Constance H Katelaris, Richard Lockey, Hilary Longhurst, William Lumry, Markus Magerl, Immaculada Martinez-Saguer, Bruce Ritchie, Alexander Nast, Ruby Pawankar, Bruce Zuraw, Marcus Maurer

**Affiliations:** 1 Department of Medicine, Pediatrics and Graduate Studies, Penn State University, Hershey, PA; 2 Center for Pediatric and Juvenile Medicine, J.W. Goethe University, Frankfurt/(Main), Germany; 3 Department of Dermatology, Johannes Gutenberg University Mainz, Mainz, Germany; 4 Departments of Medicine and Pediatrics, University of Calgary, Calgary, Canada; 5 HAEi, Orsay, France; 6 3rd Department of Internal Medicine, Semmelweis University, Budapest, Hungary; 7 Outpatient Group of Recurrent Infections and Laboratory of Immunology, Faculty of Medicine ABC; Department of Dermatology, Faculty of Medicine, University of São Paulo, São Paulo, Brazil; 8 Department of Medicine, Campbelltown Hospital, University of Western Sydney, Sydney, New South Wales, Australia; 9 Allergy, Asthma and Immunology Associates of Tampa Bay, University of South Florida, Tampa, FL; 10 Department of Immunology, Barts Health NHS Trust, London, United Kingdom; 11 Allergy and Asthma Specialists, Dallas, TX; 12 Department of Dermatology and Allergy, Allergie-Centrum-Charité, Charité-Universitätsmedizin Berlin, Berlin, Germany; 13 Department of Medicine, University of Alberta, Edmonton, Canada; 14 Division of Allergy, Dept. of Pediatrics, Nippon Medical School, Tokyo, Japan; 15 President WAO, Professor of Medicine, Department of Medicine, University of California San Diego and San Diego VA Healthcare

**Keywords:** Hereditary Angioedema, Guidelines, HAE, therapy, management, diagnosis, medications, international

## Abstract

Hereditary Angioedema (HAE) is a rare disease and for this reason proper diagnosis and appropriate therapy are often unknown or not available for physicians and other health care providers. For this reason we convened a group of specialists that focus upon HAE from around the world to develop not only a consensus on diagnosis and management of HAE, but to also provide evidence based grades, strength of evidence and classification for the consensus. Since both consensus and evidence grading were adhered to the document meets criteria as a guideline. The outcome of the guideline is to improve diagnosis and management of patients with HAE throughout the world and to help initiate uniform care and availability of therapies to all with the diagnosis no matter where the residence of the individual with HAE exists.

## Review Board Consultation Group

Richard Gower, http://rgower@marycliffallergy.com (United States)

Aleena Banerji (United States)

Marc Riedel (United States)

Paula Busse (United States)

Paul Potter (South Africa)

Yuxiang Zhi (China)

Reshef Avner (Israel)

Dumitru Moldovan (Romania)

Andrew MacGinnitie (United States)

Mark Gompels (United Kingdom)

Wolfhart Kreuz (Germany)

Laurence Bouillet (France)

Peter Spaeth (Switzerland)

Wei Te Lei (Taiwan)

William Smith (Australia)

Hiok Hee Chng (Singapore)

Kazuo Akiyama (Japan)

Werner Aberer (Austria)

Isabelle Boccon-Gibot (France)

Teresa Caballero (Spain)

Dorottya Csuka (Hungary)

Gullermo Guidos-Fogelbach (Mexico)

Allen Kaplan (United States)

Alex Malbran (Argentina)

Juan Matta Campos (Mexico)

Sandra Nieto (Mexico)

Nieves Prior (Spain)

Elias Toubi (Israel)

Lillian Varga (Hungary)

Andrea Zanichelli (Italy)

## Voting member societies of the World Allergy Organization (October 2012)

Albanian Society of Allergology and Clinical Immunology

Allergy & Immunology Society of Sri Lanka

Allergy and Clinical Immunology Society (Singapore)

Allergy Society of South Africa

Allergy, Asthma and Immunology Society of Thailand

American Academy of Allergy, Asthma and Immunology

American College of Allergy, Asthma and Immunology

Argentine Association of Allergy and Clinical Immunology

Argentine Society of Allergy and Immunopathology

Australasian Society of Clinical Immunology and Allergy

Austrian Society of Allergology and Immunology

Azerbaijan Society for Asthma, Allergy and Clinical Immunology

Brazilian Society of Allergy and Immunopathology

British Society for Allergy and Clinical Immunology

Bulgarian National Society of Allergology

Canadian Society of Allergy and Clinical Immunology

Colombian Allergy, Asthma, and Immunology Association

Croatian Society of Allergology and Clinical Immunology

Cuban Society of Allergology

Czech Society of Allergology and Clinical Immunology

Danish Society for Allergology

Dutch Society of Allergology

Egyptian Society of Allergy and Clinical Immunology

Egyptian Society of Pediatric Allergy and Immunology

Finnish Society of Allergology and Clinical Immunology

German Society for Allergology and Clinical Immunology

Honduran Society of Allergy and Clinical Immunology

Hong Kong Institute of Allergy

Hungarian Society of Allergology and Clinical Immunology

Icelandic Society of Allergy and Immunology

Indian College of Allergy, Asthma and Applied Immunology

Indonesian Society for Allergy and Immunology

Israel Association of Allergy and Clinical Immunology

Italian Society for Allergology and Clinical Immunology

Japanese Society of Allergology

Jordanian Society for Allergy and Clinical Immunology

Korean Academy of Allergy, Asthma and Clinical Immunology

Kuwait Society of Allergy and Clinical Immunology

Latvian Association of Allergists

Lebanese Society of Allergy and Immunology

Malaysian Society of Allergy and Immunology

Mexican College of Pediatricians Specialized in Allergy and Clinical Immunology Mongolian Society of Allergology

Norwegian Society of Allergology and Immunopathology

Panamanian Association of Allergology and Clinical Immunology

Philippine Society of Allergy, Asthma and Immunology

Polish Society of Allergology

Romanian Society of Allergology and Clinical Immunology

Russian Association of Allergology and Clinical Immunology

Slovenian Association for Allergology and Clinical Immunology

Spanish Society of Allergology and Clinical Immunology

Swiss Society of Allergology and Immunology

Turkish National Society of Allergy and Clinical Immunology

Uruguayan Society of Allergology

### Contributing Regional Member Societies

American Academy of Allergy, Asthma and Immunology

American College of Allergy, Asthma and Immunology

Asia Pacific Association of Allergy, Asthma and Clinical Immunology

European Academy of Allergy and Clinical Immunology

Latin American Society of Allergy and Immunology

Hereditary angioedema (HAE) is a global health problem, and evidence-based guideline recommendations are needed to inform and guide clinical decision makers. This document presents the first global guideline for the management of HAE and was developed by the World Allergy Organization (WAO) HAE International Alliance. The WAO guideline on the management of HAE differs from previous consensus reports and position papers. It results from a complete review of the underlying evidence based on systematic and transparent assessments of the quality of this evidence in evidence profiles. Furthermore, we used a Grading of Recommendations Assessment, Development and Evaluation (GRADE)-based approach for developing the recommendations provided by this guideline [1]. GRADE is recommended by the World Health Organization and takes into account that evidence alone is insufficient and that values and preferences, clinical circumstances, and clinical expertise inevitably influence decisions.

During the planning of the WAO HAE International Guidelines, Dr Richard Lockey, then President of the WAO, and Dr Timothy Craig, Chair of the committee, requested nominations from WAO-Affiliated Allergy and Immunology Associations to appoint members to the steering committee.

In the development of this guideline, the Steering Committee first agreed, during a consensus meeting in September 2010 in Gargnano, Italy, on the clinical questions to be addressed by the guideline. Working groups were assigned to review and assess the evidence available to answer these questions [2].

Based on the assessment of the evidence, the panel members, in a consensus conference at the European Academy of Allergy and Clinical Immunology (EAACI) Annual Meeting in June 2011 in Istanbul, Turkey, developed recommendations. Strong recommendations indicate that most physicians would want and only few would not want the recommended course of action, that adherence to the recommendation in clinical practice could be used as a quality criterion, and that the recommendation can be adapted as policy in most situations. Weak recommendations should be interpreted to indicate that the majority of physicians would want, but that many would not want, the suggested course of action, that in clinical practice, different choices will be appropriate for individual patients, and that policy making will require substantial debate and the involvement of various stakeholders. Understanding the interpretation of these 2 grades--either strong or conditional--of the strength of recommendations is essential for clinical decision making.

A second uniqueness of this document is that world involvement was ensured by requesting nominations for the committee from affiliated Allergy and Immunology Associations of the WAO. This worldwide approach was necessary to have global appeal and opinion in the care of HAE. Even though therapies are limited in certain areas of the world, it is hoped that this consensus will help justify the use of therapies for all patients. Furthermore, this consensus hopefully will assist clinicians, medical associations, HAE patient associations, and national medical communities to appeal for the appropriate therapies for all patients with HAE.

The goal of this guideline is to provide clinicians and their patients with guidance for rational decisions in the management of HAE types 1 and 2 (HAE-1/2). To this end, 20 recommendations [numbered and given in framed boxes] were developed. The key clinical questions covered by these recommendations are (1) How should HAE be defined and classified? (2) How should HAE be diagnosed? (3) Should HAE-1/2 patients receive prophylactic and/or on-demand treatment and what treatment options should be used? (4) Should HAE-1/2 management be different for special HAE-1/2 patient groups such as pregnant/lactating women or children? and (5) Should HAE-1/2 management incorporate self-administration of therapies and patient support measures?

## Materials and methods

### Nomination of Experts

Individuals were nominated to the expert panel and group of authors by the WAO and affiliated associations. At least one of the following criteria had to be fulfilled: (1) extensive clinical experience in the treatment of HAE, (2) relevant publications in the field of HAE, and (3) relevant experience in evidence-based medicine. Emphasis was place on selecting a representative panel of experts from throughout the world to ensure global expertise and consensus. In addition, the WAO requested a representative from the international HAE patient association (HAEi) to participate as a steering committee member. Nine patient representatives were nominated by HAEi and participated in the process (selection of key questions and consensus conference) [2].

### Support for the Consensus

The consensus conference held in Gargnano del Garda, Italy, from September 26 to 29, 2010, hosted 58 experienced HAE expert physicians from 17 countries (listed as Hereditary Angioedema International Working Group). In addition, there were 22 representatives of the 5 pharmaceutical companies producing drugs for HAE. The latter were invited to provide additional data on their products. These representatives did not take part in any voting procedures [2].

Four of the 5 pharmaceutical companies producing drugs for HAE (CSL Behring, Marburg, Germany; Dyax, Cambridge, MA; Pharming, Leiden, the Netherlands; Shire, Dublin, Ireland; and ViroPharma, Exton, PA) provided the financial support for the document and required meetings. Pharming did not have a product on the market at the time of the initiation of this endeavor. No company was present during the meetings, had input into the manuscript, or allowed to provide feedback. The pharmaceutical companies were not allowed to view the document before publication. This was essential to prevent bias and real or perceived commercial influence on the outcomes. All participants were required to submit conflict of interest statements to participate on the committee and were also required to submit conflict of interest documents with the manuscript.

### Selection of Key Questions

At the 2010 Gargnano Consensus Conference, the guideline steering committee selected key questions by means of majority vote. Expert panel members were assigned to topic-focused working groups. These working groups performed targeted literature searches and used those results together with the outcome of the 2010 Gargnano Consensus Conference-developed guideline recommendations. These recommendations were then discussed and finalized during the 2011 Istanbul Consensus Conference. The working groups determined that for many of the key questions in HAE management, little or no evidence is available to base answers and recommendations on. The expert panel recognizes this current limitation.

### Literature Search

To find relevant literature for the identified key questions, we performed systematic searches of the MEDLINE and COCHRANE databases. Our search covered the period from January 1985 through September 2010. The search strategies were as follows, displayed in Table1

**Table 1:** Search Strategies

Step	Search Term	Hits
1	"hereditary angioedema"	832
2	"hereditary angiooedema"	10
3	"hereditary angio-oedema"	85
4	"hereditary angioneurotic edema"	292
5	"hereditary angioneurotic oedema"	91
6	1 OR 2 OR 3 OR 4 OR 5	1275
7	limit 6 to yr = "1985-Current"	894
8	limit 7 to "Clinical Trial"	33

Results of search 8: Munch and Weeke, [3] Lewis, [4] Gluszko et al, [5] Cicardi et al, [6] Waytes et al, [7] Cicardi et al, [8] Goring et al, [9] Kunschak et al, [10] Farkas et al, [11] Bork and Barnstedt, [12] Gompels et al, [13] Weiler et al, [14] Szeplaki et al, [15] van Doorn et al, [16] Bas et al, [17] Bork et al, [18] Ott et al, [19] Schneider et al, [20] Varga et al, [21] Visy et al, [22] Birjmohun et al, [23] Cedzynski et al, [24] Szegedi et al, [25] Craig et al, [26] Kreuz et al, [27] Bas et al, [28] Bernstein et al, [29] Cicardi et al, [30] Cicardi et al, [31] Craig et al, [32] Levy et al, [33] Zuraw et al, [34] Zuraw et al [35].

Reasons for excluding a study from the 33 hits of search 8 were as follows: (1) no original phase 3 (or 4) trials for acute treatment of HAE, (2) no human data, (3) no clinical data, and (4) not dealing with the management of HAE/not suitable to answer selected key questions. The bibliographic information of the included trials was transferred to an endnote database, and full-text publications were obtained. Further analysis was done using a standardized Literature Evaluation Form. Evaluation was done by 2 independent assessors, who had been trained in using the Literature Evaluation Form. Any discrepancies were reviewed by a third assessor and resolved through discussion.

**Figure 1:**
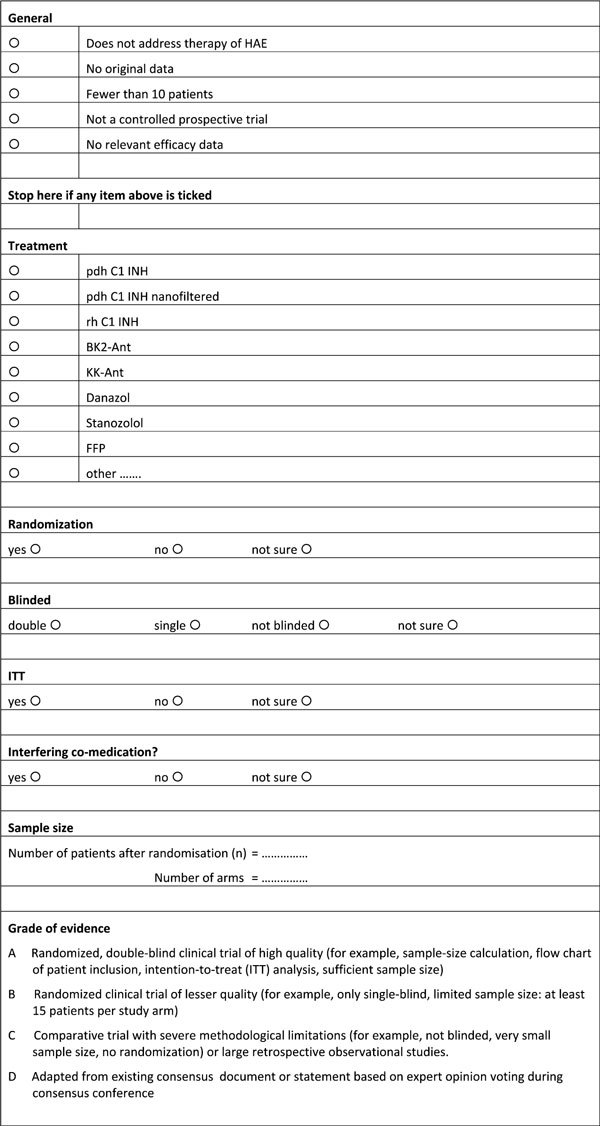
**Literature evaluation form**.

Using the Literature Evaluation Form (Figure 1), the methodologists evaluated a total of 33 studies. Of these studies, 8 phase 3 trials were ultimately included [7,10,26,30,31,33-35].

To further facilitate the evidence review, we also searched for meta-analyses, guidelines, and consensus statements, as seen in Table2

**Table 2:** Searching for meta-analyses, guidelines and consensus statements

Step	Search Term	Hits
9	Limit 7 to "Meta-Analysis"	0
10	Limit 7 to "Practice Guideline"	0
11	Limit 7 to "Guideline"	0
12	7 AND "consensus"	12

Results of search 12: Bissler et al, [36] Zhang et al, [37] Bowen et al, [38] Bowen et al, [39] Bracho, [40] Chinen and Shearer, [41] Gompels et al, [42] Bowen et al, [43] Bowen, [44] Bowen et al, [45] Bowen et al, [46] and Longhurst et al [47].

Four out of 12 (search 12) identified international consensus articles were considered relevant and were distributed to the group as a basis for discussion and adaptation [39,43,46,47].

To answer selected key questions, the search was further specified to the following, seen in Table3

**Table 3:** Further specifying the search

Step	Search Term	Hits
13	8" AND danazol[ti] OR stanozolo[ti] OR tibolone[ti]	6

Results of search 13: Cicardi et al, [8] Farkas et al, [11] Szeplaki et al, [15] Ott et al, [19] Birjmohun et al, [23] and Szegedi et al [25].

Reasons for excluding a study were as follows: (1) no original data, (2) no human data, (3) no clinical data, and (4) not dealing with the management of HAE/not suitable to answer selected key questions. After screening, all hits fulfilled the inclusion criteria.

### Grade of Evidence

Each trial included in the guideline was evaluated with regard to its methodological quality and assigned a grade of evidence according to the grading system used in previous guidelines: A, Randomized, double-blind, clinical trial of high quality (eg, sample size calculation, flow chart of patient inclusion, intention-to-treat analysis, sufficient sample size); B, Randomized clinical trial of lesser quality (eg, only single blind and limited sample size: at least 15 patients per study arm); C, Comparative trial with severe methodological limitations (eg, not blinded, very small sample size, and no randomization) or large retrospective observational studies; D, Adapted from existing consensus document or statement based on expert opinion voting during consensus conference.

### Strength of Recommendation

To avoid any potential confusion, standardized phrases were used to express the strength of a recommendation throughout the guideline. The following GRADE definitions for recommendation strength were used: Strong recommendations indicate that the desirable effects of an intervention clearly outweigh the undesirable effects. When the trade-offs were less certain, a "conditional" strength of recommendations was assigned. In case of strong expert support, a strong recommendation was applied even in cases where only very little evidence was available.

### Classification of Evidence

A level of evidence was assigned to each clinical question based on the available trials/articles. The following levels of evidence for relevant key questions were applied; low levels of evidence indicate a need for more high-quality research in these areas. See Table4

**Table 4:** Levels of Evidence for Key Questions

Grade of Evidence	Number of Trials	Authors
A	6	Craig et al, [26] Cicardi et al, [30] Cicardi et al, [31] Levy et al, [33] Zuraw et al, [34] Zuraw et al [35]
B	1	Kunschak et al [10]
C	7	Waytes et al, [7] Cicardi et al, [8] Farkas et al, [11] Szeplaki et al, [15] Ott et al, [19] Birjmohun et al, [23] Szegedi et al [25]
D	4	Bowen et al, [39,43,46] Longhurst et al [47]

### Final Consensus Conference

Based on the assessment of the evidence, the panel members, during a consensus conference in June 2011 in Istanbul, Turkey, the expert group developed recommendations and agreed on the strength of these recommendations. International experts in HAE (the review board consultation group) were requested to review the Guidelines and provide feedback, suggestions and areas of dispute with the authors. The guidelines were then reviewed by the official body of the WAO composed of the regional allergy societies and the content and document were approved by the House of Delegates as the international guideline for HAE.

## Definitions, Nomenclature, and Classification

Angioedema is defined as a vascular reaction of the deep dermis or subcutaneous/submucosal tissues with localized dilatation and increased permeability of blood vessels resulting in tissue swelling [48-52]. Angioedema can be mediated by bradykinin or mast cell mediators including histamine (Table 5) [53]. Bradykinin-mediated angioedema can occur either on a hereditary or acquired basis, due to a deficiency/defect of C1 inhibitor (C1-INH) or not (Table 5) [54-56]. Three forms of HAE have been defined: (1) HAE due to C1-INH deficiency (type 1 HAE, HAE-1), characterized by low antigenic and functional C1-INH levels; (2) HAE due to C1-INH dysfunction (type 2 HAE, HAE-2), characterized by normal (or elevated) antigenic but low functional C1-INH levels; and (3) HAE with normal C1-INH antigenic and functional levels (HAE-3). Acquired C1-INH deficiency refers to patients with angioedema due to C1-INH deficiency on an acquired basis [39,46,57]. There are a variety of acquired types of angioedema not due to C1-INH deficiency, and these may be bradykinin mediated [eg, angiotensin-converting enzyme inhibitor (ACE-I)-induced angioedema] or mast cell mediator histamine mediated (eg, urticarial angioedema, anaphylactic angioedema).

**Table 5:** Classification of Angioedema

Bradykinin-Induced AE				Mast Cell Mediator-Induced AE		
		
C1-INH Deficiency/Defect		C1-INH Normal		IgE Mediated	Non-IgE Mediated	Unknown Mediator Idiopathic AE
Inherited	Acquired	Inherited	Acquired	Angioedema with anaphylaxis Urticaria	Angioedema with urticaria	--
HAE-1	AAE	HAE-3	ACE-I	--	--	--
HAE-2						

AAE, acquired angioedema due to C1 inhibitor deficiency; ACE-I, angiotensin-converting enzyme-induced angioedema; AE, angioedema; HAE-1, hereditary angioedema due to C1 inhibitor deficiency; HAE-2, hereditary angioedema due to C1 Inhibitor defect; HAE-3, hereditary angioedema with normal C1 inhibitor levels.

## Pathophysiology

### Type 1 HAE and Type 2 HAE

HAE due to C1-INH deficiency/defect (HAE) is a rare disorder affecting approximately 1:50,000 individuals. It is transmitted in an autosomal dominant genetic pattern. HAE-1/2 are caused by a large array of different mutations of the *SERPING1 *gene, which codes for C1-INH. In approximately 20%-25%, a de novo mutation of *SERPING1 *is responsible for the disease [12,58,59].

C1-INH is a member of the serine protease inhibitor (serpin) superfamily and the major inhibitor of several complement proteases (C1r, C1s, and mannose-binding lectin-associated serine protease 1 and 2) and contact system proteases (plasma kallikrein and coagulation factor XIIa) and a relatively minor inhibitor of the fibrinolytic protease plasmin and the coagulation protease factor XIa [60-62].

Compelling laboratory and clinical data have conclusively shown that bradykinin is the primary mediator of swelling in HAE-1/2. The nanopeptide bradykinin is generated when active plasma kallikrein cleaves high-molecular weight kininogen. Plasma kallikrein is activated from its inactive zymogen by the protease factor XII, and both plasma kallikrein and factor XII are normally inhibited by C1-INH. Bradykinin has a number of important effects on the body including normal homeostasis, normal immune responses, inflammation, vascular tone, and vascular permeability. The vascular permeability-increasing effect of bradykinin in angioedema is primarily mediated through the bradykinin B2 receptor [63-65].

### Type 3 HAE

HAE with normal C1-INH (HAE-3) is a very rare disease. The symptoms are very similar to HAE-1/2. A subset of HAE-3 patients exhibits mutations in factor XII, which are thought to likely be responsible for the disease. The genetic abnormality of most HAE-3 patients has not yet been defined. Because of the lack of a clear genetic definition of this type of HAE, the diagnosis requires a family history of angioedema. There is clinical evidence that bradykinin also plays a major role in HAE-3 [66-68].

### Diagnosis

HAE-1/2 should be suspected when a patient presents with a history of recurrent angioedema, especially if wheals (hives) are absent. This suspicion is further substantiated when patients report (1) a positive family history; (2) onset of symptoms in childhood/adolescence; (3) recurrent abdominal pain attacks; (4) occurrence of upper airway edema; (5) failure to respond to antihistamines, glucocorticoid, or epinephrine; and (6) presence of prodromal signs or symptoms before swellings. Suspicion of HAE-1/2 should prompt laboratory workup as the diagnosis of HAE-1/2 requires laboratory confirmation [39,43,46,69].

#### Recommendation 1

All patients suspected to have HAE-1/2 (ie, recurrent angioedema in the absence of a known cause) should be assessed for blood levels of C4, C1-INH protein, and C1-INH function, and these tests, if abnormally low, should be repeated to confirm the diagnosis. Evidence grade: D, strength of recommendation: strong.

Measurements of serum levels of C4, C1 inhibitor protein, and the C1 inhibitor functional activity are the major laboratory tests used to diagnose HAE-1/2. In some cases, measuring the C1q level may be useful to help exclude AAE. Abnormal results should be confirmed, and normal results may need to be checked during an attack of angioedema. C4 is the single best screening test, and repeating the C4 during an attack increases the probability that a low C4 will be found. However, C4 is not a definitive test because neither the sensitivity nor specificity is absolute. There are occasional false-negative results that will be encountered, particularly in patients who are taking anabolic androgens. The C4 level should virtually always, however, be reduced during an attack of angioedema. Measurement of the activation product C4d may avoid false-negative results. False positives can also be encountered. In summary, the C4 level is useful for screening but cannot be relied upon to confirm or exclude a diagnosis of HAE-1/2 [13,70,71].

In a patient with a high index of suspicion for HAE, the C1-INH antigenic level and/or functional activity should be directly measured. The C1-INH antigenic level is low in HAE-1 and acquired C1-INH deficiency patients but is normal in HAE-2 patients. The C1-INH functional activity is low in HAE-1 and HAE-2 and acquired C1-INH deficiency patients [13,42,72].

In rare patients, sequencing of the *SERPING1 *gene can be done to pursue diagnosis of HAE-1/2; sequencing of factor XII genes can help to diagnose HAE-3; however, it is rare that this approach is needed. Complement C3 levels are expected to be normal, and testing CH50 is not useful. Many patients with acquired C1-INH deficiency have autoantibodies that recognize and inactivate C1-INH [73-75].

### Differential Diagnosis

**Figure 2:**
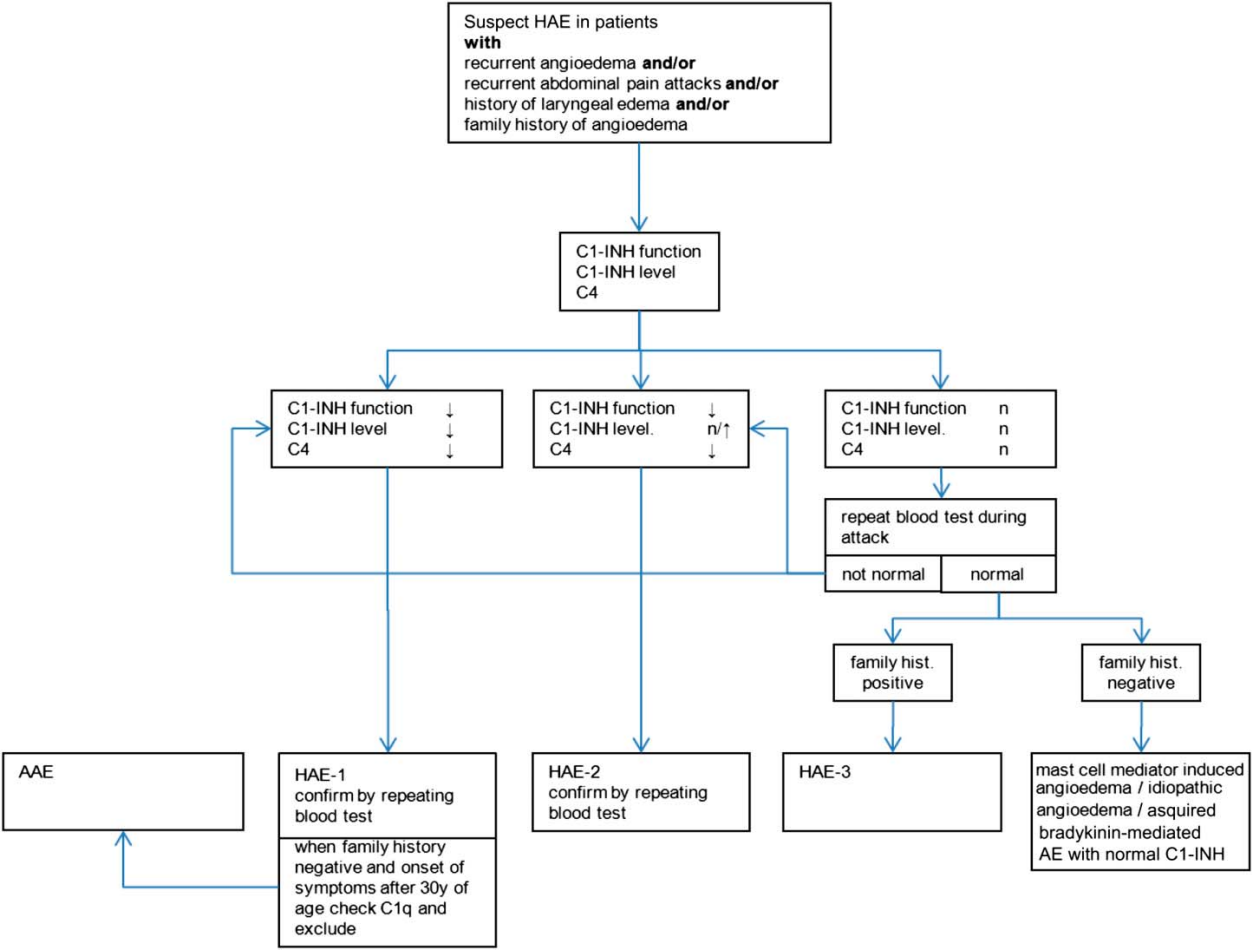
**Diagnostic algorithm for HAE-1/2**. AAE = Acquired angioedema due to C1-INH deficiency.

The differential diagnoses of HAE-1/2 include HAE-3 (ie, HAE with normal C1-INH), acquired C1-INH deficiency (AAE), ACE-induced angioedema, mast cell mediator-induced angioedema (eg, chronic spontaneous urticaria, allergic angioedema), and idiopathic angioedema (Figure 2). Because the pathophysiology and the treatment of these diseases are different than those of HAE-1/2, it is important to determine the exact diagnosis so that appropriate therapy for the specific type of angioedema the patient is manifesting can be used [39,43,46,53,76].

Mast cell mediator-induced angioedema is frequently associated with wheal-and flare-type skin reactions (hives), ie, in patients with chronic urticaria, and is more common than bradykinin-induced angioedema. Antihistamines, epinephrine, and glucocorticosteroids are effective in treating mast cell mediator-induced angioedema, but higher than standard doses of antihistamines are frequently necessary. Because mast cell mediator-induced angioedema is far more common than HAE-1/2, therapy with antihistamines and, if necessary, with epinephrine and glucocorticosteroids, is indicated when the diagnosis is not yet determined and the history seems to be inconsistent with HAE [77,78].

HAE-3 is similar to HAE-1/2 but shows differences in the underlying pathophysiological pathways. Because of this, the management may be different and it should not be assumed that HAE-3 will respond similarly to available therapies designed to treat HAE-1/2 [66,67,79]. Angioedema may be secondary to medications and frequently complicates the use of ACE inhibitors (ACE-I). One in 200 to 1000 patients treated with ACE-I will develop angioedema. Angioedema from ACE-I is bradykinin-induced but has a different mechanism than HAE-1/2. Because of the difference in pathophysiology, response to medications used for HAE-1 and HAE-2 cannot be assumed to work in ACE-I angioedema.

Angioedema due to acquired C1-INH deficiency is a rare disease that presents similarly to HAE. Differences include onset in later age, often underlying diseases such as lymphoma or monoclonal gammopathy, occasional constitutional symptoms, and depressed C1 q-r-s levels. C1q level measurements should be obtained to investigate patients for AAE-C1-INH, especially with new onset of angioedema after the age of 40 years [80-82].

## Therapy of HAE-1/2

### On-Demand Treatment

#### Recommendation 2

All attacks that result in debilitation/dysfunction and/or involve the face, the neck, or the abdomen should be considered for on-demand treatment. Treatment of attacks affecting the upper airways is mandatory. Evidence grade: D, strength of recommendation: strong.

#### Recommendation 3

We recommend that attacks are treated as early as possible. Evidence grade: D, strength of recommendation: strong.

Attacks of the upper airways can result in asphyxiation. Abdominal attacks are painful and debilitating. Peripheral attacks, for example, of hands or feet, result in impaired function. All of these consequences of HAE-1/2 attacks can be minimized by on-demand treatment. Evidence suggests that early treatment is advantageous. Every patient with HAE-1/2 should be considered for home therapy and self-administration training, once the diagnosis of C1-INH deficiency is confirmed [47,83-86].

#### Recommendation 4

We recommend that HAE attacks are treated with C1-INH, ecallantide, or icatibant. Evidence grade: A, strength of recommendation: strong.

**Figure 3:**
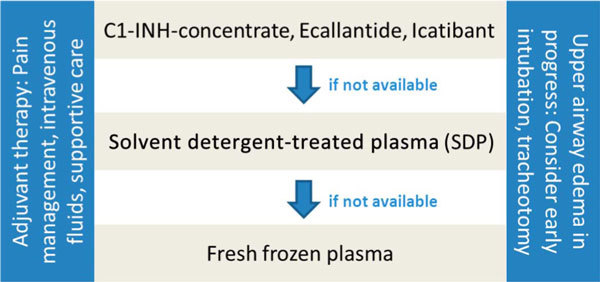
**Treatment algorithm for HAE-1/2**.

If these drugs are not available, attacks should be treated with solvent detergent-treated plasma (SDP). If SDP is not available, then attacks should be treated with frozen plasma (where safe supply is available). Evidence grade: D, strength of recommendation: strong (Figure 3; Table 7).

#### Recommendation 5

We recommend that intubation or tracheotomy is considered early in progressive upper airway edema. Evidence grade: D, strength of recommendation: strong.

#### Recommendation 6

We recommend that patients with attacks receive adjuvant therapy when indicated (pain management, intravenous fluids, and supportive care), but specific therapies should be used without delay when indicated. Evidence grade: D, strength of recommendation: strong.

#### Recommendation 7

We recommend that oral antifibrinolytics are not to be used as on-demand treatment. Evidence grade: A, strength of recommendation: strong.

#### Treatment With C1-INH Concentrate

Treatment with C1-INH concentrate eliminates the underlying cause of HAE-1/2 by replacing the deficient protein. Exogenous C1-INH concentrate acts on the same targets as endogenous C1-INH and inactivates C1r, C1s, and mannose-binding lectin-associated serine proteases in the complement system and factor XII, factor XI, and thrombin in the coagulation cascade and kallikrein in the contact system and tissue-type plasminogen activator and plasmin in the fibrinolytic system. Treatment results in an increase of the plasma levels of the C1-INH and helps to regulate activation of all cascade systems involved in the production of bradykinin released during attacks [7,10,26,32,35].

#### Plasma-Derived C1-INH

Plasma-derived C1-INH (pdC1-INH) is obtained from human blood. One unit of pdC1-INH is equivalent to the C1-INH content of 1 mL of human plasma (270 mg/L).

Three pdC1-INH concentrates are approved for on-demand treatment of HAE-1/2 in Europe: Berinert (CSL Behring), Cetor (Sanquin, Amsterdam, the Netherlands), and Cinryze (Viropharma) and one in the United States: (Berinert; CSL Behring). Berinert and/or Cinryze are also available in many other countries, for example, both drugs are registered for use in Australia.

Berinert [20 U/kg body weight slowly intravenous (IV)] is approved for the treatment of all types of attacks of HAE-1/2 in adults, children, and neonates. Cetor/Cinryze (1000 U slowly IV) are indicated for all types of attacks for ages 12 years and over.

pdC1-INH is obtained by separating C1-INH from cryodepleted human plasma by adsorption and precipitation, purification, pasteurization, and nanofiltration. The plasma half-life of pdC1-INH is longer than 24 hours (Berinert: 32-47 hours; Cetor/Cinryze: 48 ± 10 hours). All pdC1-INH products are comparable in efficacy and adverse effect profile. The safety and tolerability of pdC1-INH are good, and few adverse events have been reported. The risk of allergic reactions is negligible. Modern pdC1-INH use has not been associated with transmission of hepatitis B or C or human immunodeficiency viruses [29,87,88].

Thrombotic events have occurred in off-label use in premature neonates receiving extremely high doses of pdC1-INH (500 U/kg body weight) to relieve capillary leak syndrome. There are no known interactions with other medicinal products. Tachyphylaxis does not appear to occur; however, some have seen increasing doses required to prevent attacks when C1-INH is used regularly for prophylaxis [89-91].

#### Recombinant C1-INH

The mode of action of recombinant human C1-INH (rhC1-INH) is identical to that of pdC1-INH. The only rhC1INH available is Ruconest (Pharming). RhC1-INH (50 U/kg body weight slowly IV) is indicated for the on-demand treatment of all types of HAE attacks in adults. It is expressed in the mammary gland of transgenic rabbits and is obtained from their breast milk. One unit of rhC1-INH corresponds to the mean quantity of C1-INH present in 1 mL fresh normal plasma. Its half-life is 3 hours. Before initiating treatment with rhC1-INH, patients must be tested for the presence of immunoglobulin E (IgE) antibodies against rabbit allergens using a validated test for IgE antibodies against rabbit epithelium (dander). IgE antibody testing should be repeated annually or after 10 treatments, whichever occurs first, although sensitization or induction of significant rabbit or rhC1-INH-related antibodies has not been seen to date. It must not be used in patients with a preexisting known or suspected allergy to rabbit dander. The most common side effect of rhC1-INH (seen in 1-10 patients out of 100) is headache and is unlikely related to the drug. Transmission of human viruses is not a concern [34,92].

#### Treatment With Kallikrein Inhibitor

Inhibition of kallikrein activity inhibits the cleavage of high-molecular weight kininogen to bradykinin and inhibits edema progression in HAE-1/2 attacks (Figure 2). The only commercial kallikrein inhibitor is ecallantide (Kalbitor; Dyax). Ecallantide (30 mg, subcutaneous (s.c.) injection) is indicated for the on-demand treatment of all types of HAE attacks in patients 16 and older. Ecallantide is a 60-amino acid recombinant protein produced by expression in the yeast *Pichia pastoris*, and has a plasma half-life of 2 hours. Because the size of the peptide and that it is foreign antibodies from both IgG and IgE classes have been detected. In addition antibodies have been demostrated to the yeast that is used in developing ecallantide [31,33,93].

#### Treatment With Bradykinin Receptor Antagonist

Bradykinin induces angioedema by activating bradykinin B2 receptors, and it is blocked by the B2 receptor antagonist, Icatibant, which prevents binding as a competitive antagonist. Icatibant (Firazyr; Shire) is a 10-amino acid synthetic peptide, which is a specific and selective competitive antagonist of the bradykinin B2 receptor and the only commercial B2 receptor antagonist. Icatibant (30 mg, s.c.) is indicated for the on-demand treatment of all types of HAE attacks in adults. It has a plasma half-life of 1 to 2 hours. The safety and tolerability of Firazyr are good. Transient local injection site reactions (erythema, wheal, pruritus, and burning sensation) occur in almost 100% of patients. Allergic reactions have not been reported [18,30,94].

##### Recommendation 8

We recommend that all patients should have on-demand treatment for 2 attacks. Evidence grade: D, strength of recommendation: strong.

##### Recommendation 9

We recommend that all patients should carry their on-demand treatment at all times. Evidence grade: D, strength of recommendation: strong.

### Short-term/Preprocedural Prophylaxis

Physical trauma and emotional stress are inevitable consequences of surgery and are well-recognized precipitants of HAE-1/2 attacks. Swellings associated with surgery usually occur 4 to 30 hours after the event. Because swellings usually occur near the site of surgical trauma, dental surgery is considered particularly high risk because of its association with upper airway edema [39,42,43,46,76,95].

Despite the perceived benefits of preprocedural prophylaxis, evidence for its efficacy is lacking. Case reports and series suggest that despite prophylaxis, swellings may occur even after relatively minor procedures. However, several reports document a reduction in the incidence of swelling for both adults and children with prophylaxis. The decision to give prophylaxis before a procedure depends on the patient's personal history and the likely risk associated with the procedure. A history of frequent swellings; swellings after a similar procedure, dental, or other intraoral surgery; the need for intubation; or more invasive procedures favor preprocedural prophylaxis. Short-term prophylaxis should also be considered to cover periods of high risk for attacks when there is either increased likelihood of attack or increased consequence of attack (ie, stressful periods, examinations, or similar).

For lower risk procedures or where safe on-demand agents are available, prophylaxis may be omitted. The patient should be aware of the risk and have a management plan and on-demand treatment for attacks. Where available, 2 doses of C1-INH concentrate, ecallantide, or icatibant, should be immediately accessible [11,95-99].

#### Recommendation 10

The administration of short-term prophylaxis should be considered before surgeries, especially dental/intraoral surgery, where endotracheal intubation is required, where upper airway or pharynx is manipulated, and before bronchoscopy or endoscopy. Evidence grade: D, strength of recommendation: strong.

For short-term/preprocedural prophylaxis, C1-INH concentrate should be used (dose yet to be fully investigated with recommendations varying from 10 to 20 U/kg body weight or 1000 U), 1 to 6 hours before procedure.

Prophylactic C1-INH concentrate dosing requires further studies. Even at 1000 units, breakthrough attacks occurred, suggesting that higher doses are necessary to fully prevent attacks. SDP can be used if C1-INH concentrate is not available.

Androgens (danazol, stanozolol) may be used for short-term/preprocedural prophylaxis when the surgery-related risk is relatively low and when C1-INH concentrate is not available. Advantages are ease of use, good tolerability for most, including children, and low cost. Disadvantages are perceived inferior efficacy to C1-INH concentrate (although evidence is lacking), use in case of elective surgery only, side effects, and unsuitability for pregnant (except last trimester) or breastfeeding women. Side effects in short-term use may include menstrual disturbance, vaginal dryness or irritation, and emotional lability. Beyond pregnancy testing, other blood tests or safety monitoring are not necessary. Very frequent short courses may lead to side effects associated with long-term use. For short-term/preprocedural prophylaxis, androgens are used for 5 days before and 2 to 5 days post event (danazol is used at 2.5-10 mg·kg^-1^·d^-1^, maximum 600 mg; stanozolol is used at 4-6 mg/d).

Tranexamic acid (TA) has been used for short-term/preprocedural prophylaxis in the past, the recommended dose for oral TA (not fully established) is 25 mg/kg 2 to 3 times daily with maximum 3 to 6 g daily. The efficacy in suppressing breakthrough attacks seems to be low [39,42,43,46,76].

### Long-term Prophylaxis of HAE-1/2

Long-term prophylaxis of HAE refers to the use of regular medication to prevent episodes of angioedema in patients with confirmed HAE-1/2. Long-term prophylaxis should be considered in all severely symptomatic HAE-1/2 patients taking into consideration the severity of disease, frequency of attacks, patient's quality of life, availability of resources, and failure to achieve adequate control by appropriate on-demand therapy. C1-INH concentrate or androgens can be used for long-term prophylaxis and the decision to use one over the other should depend upon contraindications, adverse events, risk factors for adverse effects, tolerance, response to intervention, and dose required to control attacks. None of the current prophylactic modalities are capable of preventing upper airway edema with certainty [39,42,43,46,76].

pdC1-INH can be used for the prevention of HAE attacks. The food and Drug Administration (FDA, USA) and the European Medicines Agency (EMA, EU) approved the prophylactic use of pdC1-INH (Cinryze) [35].

Dosing should be twice a week based upon the half-life of C1-INH. Dose and/or frequency may need adjustment for optimum control. On-demand therapy (C1-INH concentrate, ecallantide, or icatibant) should be available for all patients on prophylaxis because breakthrough attacks occur in most patients on long-term prophylaxis [35,99-101].

Vaccination with hepatitis A and B and serologies to these viruses and to hepatitis C, hepatitis E, human immunodeficiency virus (HIV), human T-lymphotropic virus, and parvovirus should be done before initiation of pdC1-INH long-term prophylaxis and once annually [42].

Long-term prophylaxis with androgen derivatives is effective but must be regarded critically, especially on account of their androgenic and anabolic effects. Most importantly, androgens can cause hepatitis in a dose-dependent manner. Therapy with androgen derivative can be hepatotoxic and can affect serum lipid levels. Virilization is the primary complication occurring in women: menstrual disorders and even amenorrhea, diminished libido, acne, and psychological disorders such as depression and aggression are also common. In addition, androgens are subject to numerous contraindications and show interactions with a large number of other drugs. Careful surveillance is imperative in preventive androgen therapy in HAE-1/2 given the number and severity of adverse effects. In addition to clinical tests and examinations and questioning of the patient, semiannual blood and urine tests are needed, and at least once a year, an ultrasound of the liver should be performed. The goal of prophylaxis with androgens is the reduction of the severity and frequency of swelling events as far as possible with a reasonable dosing scheme [102-110].

#### Recommendation 11

Before the initiation of long-term prophylaxis with androgens, measurements of complete blood count, urine analysis, liver function tests, lipid profile, assessment of cardiac risk factors, and liver ultrasound should be performed. While using androgens for long-term prophylaxis and for 6 months after stopping therapy, complete blood count, urine analysis, lipid panel, liver function tests, and blood pressure should be monitored every 6 months and an ultrasound of the liver should be done yearly to assess for adverse events associated with androgens and contraindications to androgens. Evidence grade: C, strength of recommendation: strong.

The dose of androgens needed to control HAE attacks can vary between the equivalent of 100 mg every other day and 200 mg of danazol 3 times a day. Dosages above 200 mg a day of danazol daily for extended periods of time are not recommended. It is suggested to commence with a moderate dose (eg, 100-200 mg daily) and increase or decrease on a monthly basis depending on the frequency of episodes until satisfactory control is reached. The response to androgens varies considerably, and the dose required for long-term prophylaxis is highly variable. For this reason, the dosage should be adjusted to clinical response and not adjusted by laboratory results [39,43,46,76].

Antifibrinolytics are not recommended for long-term prophylaxis because data supporting their efficacy are lacking. Nevertheless, they are widely used especially when androgens are contraindicated and may anecdotally have some benefit in a minority of patients. Side effects are usually minor. They include gastrointestinal upsets (can be reduced by taking the drug with food), myalgia/creatine kinase elevation, and a theoretical risk of thrombosis. Contraindications/precautions include presence of thrombophilia or increased thrombotic risk or acute thrombosis. The recommended dose of TA is 30 to 50 mg/kg daily in 2 to 3 divided doses, to a maximum of 6 g, but dose ranging studies or blinded studies comparing with other antifibrinolytics placebo, androgens, or C1-INH have not been performed [30,39,43,111-114].

## Management of HAE-1/2 in children

### Course of Disease and Clinical Picture

HAE-1/2 symptoms may occur at any age, but attacks usually begin during school-age years or adolescence. In 50% of cases, the initial onset of symptoms occurs between 5 and 11 years. The gene defect is present at birth; however, symptoms are uncommon during neonatal age or infancy. Subcutaneous edema is the most common and the earliest symptom. Upper airway edema rarely is an initial manifestation. Asphyxia may ensue more rapidly in children because of smaller airway diameter. The earliest occurrence was described in a 3-year-old patient. Estimating the prevalence of abdominal attacks in the pediatric population is difficult, because "belly ache" is a common symptom, especially in infants. The frequency and the severity of symptoms may increase during adolescence. The earlier the onset of symptoms, the more severe the subsequent course of HAE-1/2.

Erythema marginatum, as a prodromal sign, is more frequent in the pediatric population. It has been observed in 42% to 58% of cases, and it is often mistaken for urticaria [115-118].

### Diagnosis

According to the Mendelian rules of autosomal inheritance, the offspring of a patient with HAE-1/2 stands a 50% chance of inheriting the disease. Therefore, it is important to establish the diagnosis as early as possible, ideally before the onset of clinical manifestations.

Prenatal diagnosis of HAE-1/2 has not become widespread in clinical practice. Reasons include (1) that no mutation of the C1-INH gene can be detected in 8% to 10% of cases, (2) that identical mutations may be associated with substantially different phenotypes, (3) that C1-INH mutations may cause a nonfatal manageable disease in the offspring, the severity of which cannot be predicted in advance, and (4) recent advances in therapy have significantly improved quality of life of patients with HAE [116,118,119].

It is not recommended to screen children with a positive family history (mother or father with HAE-1/2) immediately after birth, because test results may be false.

After birth, complement protein levels exhibit a continuous increase--which, however, follows a nonuniform pattern--and reach the normal value by the age of 0.5 to 1 year. Complement concentrations measured in cord blood of full-term neonates are lower than maternal levels. Antigenic and functional C1-INH levels correspond to 70% and 62% of adult values, respectively. Therefore, testing under 1 year of age may not be reliable and should be confirmed after age 1 [120-122].

#### Recommendation 12

Screening children for HAE-1/2 should be deferred until the age of 12 months, and all offspring of an affected parent should be tested. Evidence grade: D, strength of recommendation: strong.

C4 and C1-INH functional level testing is advisable in all children with isolated angioedema of unknown etiology. Genetic testing can prove helpful but is rarely necessary or suggested.

### Therapy

Similar to adults, all pediatric HAE patients need a treatment action plan and on-demand therapy.

#### Recommendation 13

The preferred on-demand therapy for HAE-1/2 attacks in children is pdC1-INH. Evidence grade: D, strength of recommendation: strong.

pdC1-INH concentrate is the only approved drug for HAE-1/2 on-demand treatment in childhood (in European Union only, 12 years and older in the United States). Treatment with pdC1-INH concentrate is effective in pediatric patients. It is well tolerated and has a good safety profile. Dosing of the pdC1-INH Berinert is 20 U/kg. The pdC1-INH Cinryze (1000 U per treatment) is licensed for the use in postpubertal adolescents in some countries. During abdominal attacks, parenteral fluid replacement may be required in view of the increased susceptibility of children to hypovolemia and because extravasation into the peritoneal cavity and the intestinal lumen can be substantial. SDP is preferred over fresh frozen plasma (Figure 3), but either should be used only as second-line treatment when first-line treatment (pdC1-INH concentrate) is not available. Recombinant C1-INH, ecallantide, and icatibant are not licensed for use in children, and experience with these drugs in this patient population is very limited [116,118,119,123].

Pediatric surgical interventions are less common and shorter in duration although dental procedures should be considered for preprocedural prophylaxis. pdC1-INH is the first-line short-term prophylactic option, but short courses of androgens can be used as second line in the event C1-INH is not available. With either option, on-demand therapy should be available because short-term prophylaxis is not 100% effective.

The majority of children do not require long-term prophylaxis. During this period of life, on-demand therapy of attacks is preferable. The indications for long-term prophylaxis in adolescents are the same as in adults [39,43,46,76,123]. The preferred therapy for long-term prophylaxis is pdC1-INH with dose requiring further study. Routine prophylactic treatment of postpubertal adolescents with Cinryze (1000 U every 3 or 4 days) is licensed in many countries and Cetor in the Netherlands [123].

When pdC1-INH is not available for long-term prophylaxis, antifibrinolytics (20-40 mg/kg) are preferred to androgens because of their better safety profile; however, efficacy is questioned by many and data are not available supporting its use. Epsilon aminocaproic acid is less well tolerated than TA. Androgens are not recommended for long-term prophylaxis in prepubertal children, but long-term use in children has been reported, and in some cases, the benefits may outweigh the risks. Androgens (dosing danazol equivalent 2.5 mg/kg per day; 50 mg/d initial dose with subsequent reduction of the dosage interval to every other day or every third day; maximum single dose is 200 mg over 10 years of age) result in masculinization and hypogonadism in boys and menstruation irregularities in girls. Unfavorable effects on mental functions and behavior are common. Reduction in ultimate body height may occur owing to the premature closure of epiphyseal growth plates. Adverse reactions can be minimized by administering the lowest effective dose.

Although prepubertal children typically experience fewer attacks than adolescents and adults, some have frequent attacks that disrupt education and family life, and long-term prophylaxis can be beneficial in these cases. Long-term prophylaxis with attenuated androgens is not recommended, and TA may be of limited benefit. pdC1-INH is effective for treatment of children, and home on-demand therapy has been used successfully in this age group [39,43,46,76,123].

#### Primary Prevention

The incidence of trigger factors differs slightly among pediatric and adult patients. Infections and mechanical trauma are more common trigger factors during childhood. Compulsory and recommended vaccinations for children are safe, and the prevention of infections may reduce the frequency of edematous attacks. Medicinal products, which can cause edema as an adverse effect, are less frequently used in children. Treatment with ACE-I is less often necessary during childhood; however, early initiation of oral estrogen containing contraceptives is increasingly common and may trigger attacks [124-126].

#### Other Management Considerations

Providing the patient and family with appropriate information is indispensable to support them to adopt a suitable lifestyle and to avoid severe complications. Pedagogues, teachers, and health care personnel responsible for the child at kindergarten or school should receive written information on the disease. pdC1-INH for emergency use should be available at home, school, and for travel including school field trips. Vaccination for hepatitis B and A should be done if not part of a country's routine vaccine recommendation [116,118].

## Management of HAE-1/2 during pregnancy and lactation

### Course of Disease and Clinical Picture

The anatomical and physiological--primarily hormonal--changes of pregnancy may influence the manifestations of HAE and interfere with the diagnosis and the treatment of HAE. Pregnancy can either mitigate or aggravate HAE; alternatively, it may have no impact whatsoever. Infrequently, the manifestations of HAE first occur during pregnancy. Attack frequency observed during previous pregnancies is not predictive of HAE events during any subsequent pregnancy. Pregnant HAE-1/2 patients require vigilant care and meticulous monitoring by an HAE expert. Patients should be managed in close cooperation by professionals from relevant medical specialties. Labor and delivery themselves only rarely induce an attack, which may occur either during labor or within 48 hours of delivery. Close follow-up is recommended for at least 72 hours postpartum after uncomplicated vaginal delivery. Breastfeeding may be associated with an increased number of maternal edematous attacks, with abdominal symptoms and facial edema, but is still recommended [87,114,127-131].

### Diagnosis

Special procedures are required for diagnosing HAE-1/2 during pregnancy. The plasma level of C1-INH decreases during pregnancy, and it returns to normal after delivery. Therefore, repeated measurements of C1-INH levels are necessary after childbirth to confirm the diagnosis of HAE [114].

### Therapy

#### Recommendation 14

During pregnancy and lactation, pdC1-INH is the preferred therapy. Evidence grade: D, strength of recommendation: strong.

pdC1-INH is recommended as first-line therapy for pregnant or breast-feeding HAE-1/2 patients as it is safe and effective. No published experience is available with recombinant C1-INH, ecallantide, and icatibant. SDP may be used when pdC1-INH is not available and fresh frozen plasma when SDP is not available (Figure 3) [114,117].

Short-term prophylaxis is recommended, preferably with pdC1-INH, before chorionic villus sampling, amniocentesis, and induced surgical abortion. Alternatively, pdC1-INH should be available and administered immediately at the onset of edema. It is recommended to manage childbirth in the hospital setting. Routine administration of short-term prophylaxis before uncomplicated natural delivery is not recommended, but pdC1-INH should be immediately available for on-demand use. Administering pdC1-INH is recommended before labor and delivery when HAE is severe, if symptoms have been recurring frequently during the third trimester, if the patient's history includes genital edema caused by mechanical trauma, when intubation is required, and when forceps delivery or vacuum extraction is performed. Short-term prophylaxes with pdC1-INH and epidural anesthesia are recommended before a cesarean section (abdominal delivery), and intubation should be avoided if possible [39,43,46,76,114].

If long-term prophylaxis is required during pregnancy, then pdC1-INH is considered a safe and effective treatment option. Antifibrinolytics may be used only when pdC1-INH is unavailable. They should be used only if clearly needed and if the potential benefit justifies the potential risk to the fetus, especially because evidence for the efficacy of the product is lacking.

Androgens are not recommended, as these drugs cross the placenta. Their adverse effects include masculinization of the female fetus, placental insufficiency, and fetal growth retardation. Breast-feeding should be discontinued before androgens are introduced: terminating lactation itself may reduce attack frequency.

pdC1-INH is considered the best therapy for on-demand treatment, short-term prophylaxis and long-term prophylaxis when indicated during lactation. Androgens and antifibrinolytics should be avoided because they are secreted in breast milk. SDP may be used for short-term prophylaxis and on-demand therapy but is not as safe as C1-INH and is a second-line agent because of the greater risk of viral transmission and the possibility of worsening an attack [39,43,46,76,114,123].

## Patient support, home therapy and self-administration, and other management considerations

### Patient Support

Patient organizations and support groups provide help and support for HAE patients and caregivers and endorse the philosophy that all patients should have the resources to control their HAE symptoms and fulfill their potential at school, at work, and in their relationships. HAEi, the International Patient Organization for C1 Inhibitor Deficiencies, and national HAE associations have active informative Web sites for patients and health care providers. HAEi has launched a "call to action" aimed at increasing the awareness and knowledge on HAE with governments, health authorities, and health care professionals and to achieve recognition of HAE as a serious, disabling, potentially life-threatening, and chronic condition that must receive timely accurate diagnosis and effective treatment.

Patient organizations also work toward identifying and addressing unmet needs in HAE management, which include the development of safe and well-tolerated oral prophylactic and on-demand therapies, the optimization of existing long-term prophylactic and on-demand therapies (eg, by dose-ranging studies, subcutaneous prophylaxis, pediatric studies, head-to-head studies), cost/effectiveness studies, increasing the availability of modern treatment options worldwide, emphasizing the need for self-care, individual action plans, early therapy, and gene therapy research. As with most diseases, information on Web sites can be questionable; however, HAEi provides reviewed, updated, and reliable information and is considered to be a quality source for patient education [47].

### Individualized Patient Action and Treatment Plans

#### Recommendation 15

All patients with HAE should have an action plan and product available to treat an attack of HAE. Evidence grade: D, strength of recommendation: strong.

Because HAE-1/2 is an unpredictable, painful, and life-threatening condition that places a huge stressful burden on patients and their families, an individualized treatment plan should be carefully developed in partnership between the physician and the patient. Individualized treatment plans should address preventive measures and home care and self-administration and include an emergency (on demand) treatment plan. Patients should carry on-demand medication and an HAE identification card with instructions on how to use the on-demand medication in case of an attack. Patients on long-term prophylaxis, with either C1-INH or androgens, also require a acute care plan and therapy for on-demand use [46,76].

### Home Therapy and Self-administration

#### Recommendation 16

We recommend that all patients who are provided with on-demand treatment licensed for self-administration should be taught to self-administer. Evidence grade: D, strength of recommendation: strong.

#### Recommendation 17

We recommend that all patients should be provided with an HAE identification card. Evidence grade: D, strength of recommendation: strong.

Every patient with HAE-1/2 should be considered for home therapy and self-administration, once the diagnosis is confirmed. Requirement to attend a medical facility may result in delayed on-demand treatment, inappropriate therapy, and many attacks are not treated at all. This contributes to the morbidity and social and economic disadvantage associated with HAE. All patients should therefore be considered for self-administration training with either C1-INH or icatibant. In the United States, ecallantide can be given at home by a health care provider trained to treat anaphylaxis. Self-administration training should include the training of a "home therapy partner" (a family member or friend who can provide support, advice, and administration of therapy when the patient is compromised or unable or uncomfortable with self-treatment) [47,84,132-136].

Patients undertaking travel and patients living alone may experience particular difficulty in obtaining on-demand treatment administered by a physician, and they may, therefore, be more likely to present late or to leave severe attacks untreated if unable to self-administer. These patient groups are likely to gain the greatest benefit from the ability to control attacks by self-administration.

It is anticipated and demonstrated that home therapy decreases the severity and duration of attacks, reduces morbidity and disability of attacks, and can improve quality of life and productivity. In addition, the cost of care is reduced considerably by the use of home and self-therapy [137,138].

C1-INH home therapy is suitable for children with frequent or disruptive attacks, where a responsible adult is available and willing to undertake training. Experience with hemophilia suggests that it is beneficial for children to be encouraged to take an active part early in their treatment, and even at the age of 8, self-administration is possible and safe [135].

Advanced age is not a contraindication for home therapy, provided that patients and home therapy partners can safely and effectively administer treatment. Also, pdC1-INH home therapy seems safe and effective and is recommended for prophylaxis or treatment of pregnant and lactating women. In contrast, long-term prophylaxis with attenuated androgens or TA is not recommended in pregnancy. Therefore, self-administration of pdC1-INH should be considered for control of symptoms for pregnant and lactating women. Women with HAE contemplating pregnancy should consider home self-administration training [87,114,128,139].

With self-administration or home administration, it is extremely important to encourage all patients to seek care after administration of therapy for an upper airway attack. With all therapies, upper airway swelling may progress or rebound and repeat dosing may be necessary. Seeking emergency care after therapy is essential to reduce the risk of death associated with airway swelling.

### Avoidance of Triggers

A variety of conditions and events are known to trigger swelling attacks. Trauma, whether accidental or associated with dental, medical, and surgical procedures, commonly results in a swelling attack. The use of estrogen-containing oral contraceptive agents and hormone replacement therapy and antihypertensive agents containing ACE inhibitors may increase the frequency or precipitate an HAE attack. Other reported triggers include psychological stress, fatigue, febrile illness, alcohol, possibly *Helicobacter pylori *infection, and the menstrual cycle. Patients should be made aware of potentially relevant triggers of attack to reduce precipitation of attacks. Influenza vaccine may reduce upper airway infections and possibly reduce upper airway swelling. Hepatitis vaccines may reduce the risk of viral transmission that can be associated with exposure to blood products. Good dental care can reduce extractions, need for aggressive dental procedures, and prevent acute or chronic intraoral inflammation, which might reduce the threshold for attacks. Monitoring for side effects of medications is important and is outlined above [22,39,43,45,140,141].

### Expert Involvement and Checkups

#### Recommendation 18

All patients with HAE should have at least 1 annual assessment by an HAE specialist. Evidence grade: D, strength of recommendation: strong.

HAE-1/2 patients, once diagnosed, are encouraged to find a health care provider with knowledge and interest in the disease. When and where possible, care should be provided by comprehensive care clinics with expertise in HAE.

It is recommended that HAE patients have a medical evaluation at least annually. Newly diagnosed patients and those on long-term prophylaxis with attenuated androgens should be seen at 3-month intervals initially and then twice a year for 2 years. Those on androgens should continue to be seen twice a year. Evaluation at follow-up visits should include recording of type and frequency and severity of symptoms, frequency of use, and effectiveness of treatments for swelling attacks. A physical examination and appropriate laboratory evaluation should be conducted (Table 6) [39,43,45].

**Table 6:** Procedures Recommended During Long-term Care for HAE Patients

Procedures	Timing
Develop action plan	When diagnosed and reviewed annually
Provide with HAE emergency card	When diagnosed and reviewed annually
Provide with 2 doses of on demand therapy	When diagnosed
Screen family	When diagnosed
Hepatitis C, B and HIV screening	When diagnosed and annually if receiving blood products
Hepatitis A and B vaccine	When diagnosed
Assessment by an HAE specialist	Annually
Influenza vaccine	Annually
If long term androgens are used	
LFT, CBC, LP, UA	At start and every 6 mo
Assess cardiac risk factors	At start and every 6 mo
Ultrasound liver	At start and every 12 mo

CBC, complete blood count; HIV, human immunodeficiency virus; LFT, liver function tests; LP, lipid profile; UA, urine analysis.

**Table 7:** First-Line Options for On-Demand Treatment of HAE-1/2

	Route of administration	Efficacy	Safety	Limitation	Self-administration	Age, yrs	Cost
Berinert	IV	+++	+++*	+†	+	All (≥12 in the United States)	High
Cinryze	IV	+++	+++*	+†	+	≥12	High
Ruconest	IV	+++	+++	+‡	-	≥18	High
Icatibant	SC	+++	+++	+§	+	≥18	High
Ecallantide	SC	+++	+++	+||	-	> 16	High

IV, intraveneous; SC, subcutaneous.

*Blood product.

†Pretreatment vaccination recommended.

‡Pretreatment and repeated allergological testing required.

§Special attention required in acute ischemic heart disease/unstable angina and in the first week after cerebral infarction.

¶Three injections.

||Posttreatment observation/keep refrigerated.

### Family Screening

#### Recommendation 19

Family members of patients with HAE should be screened so that appropriate therapy can be available for treatment, especially because the first event may be of the upper airway and fatal without appropriate therapy. Evidence grade: D, strength of recommendation: strong.

HAE-1/2 is a genetic disorder with autosomal dominant transmission. Family members including grandparents, parents, siblings, children, and grandchildren of HAE-1/2 patients should be screened for C4, C1-INH level, and C1-INH functional plasma levels [46,69,76].

If these values are normal in family members with unexplained abdominal pain or angioedema, then these tests should be repeated at the time a swelling attack occurs [39,43,45].

### Vaccinations

#### Recommendation 20

Hepatitis A and B vaccination should be administered to HAE-1/2 patients receiving blood products, including pdC1-INH. All patients should receive influenza vaccine. Evidence grade: D, strength of recommendation: strong.

All HAE patients have a potential for receiving human blood products. Because of this, HAE patients should be screened as early as possible for hepatitis B and C and HIV. Vaccination for hepatitis A and B reduces the risk of infections and the use of blood products including pdC1INH that bears a theoretical risk of pathogen transmission. Annual assessment for infections with hepatitis B, C, and HIV is suggested [46,69,76].

## End notes

T. Craig, Research: CSL Behring, Dyax, Pharming, Shire, and Viropharma. Speaker: CSL Behring, Dyax, Shire, and Viropharma. Unrestricted Educational Grants: CSL Behring, Dyax, and Viropharma. Consultant: CSL Behring. E. Aygören Pürsün has received speaker fees from CSL Behring, Shire, and Viropharma and has served on advisory boards for CSL Behring, Pharming, Shire, and Viropharma. K. Bork: consultant of CSL Behring, Shire, and ViroPharma. T. Bowen, in the past year, has served on an Advisory Board for CSL Behring Canada. H. Boysen is an Executive Director of Hereditary Angioedema Association International. H. Farkas has received speaker fees from Shire and served on Advisory Board for CSL Behring, Shire, Pharming, and Viropharma. A. S. Grumach has served on Advisory Boards and as speaker for Shire (Brazil). Dr C. H. Katelaris has served on Advisory Boards for Shire, Australia, and CSL Behring, Australia. R. Lockey is the Past President of World Allergy Association. H. Longhurst: (research) CSL Behring, Shire, and Viropharma. Speaker: CSL Behring, Shire, SOBI, and Viropharma. Unrestricted Educational Grants: CSL Behring and Shire. Consultant: CSL Behring, Shire, and SOBI. W. R. Lumry, Consultant Arrangements: CSL Behring, Dyax, Shire HGT, and Viropharma Pharmaceuticals. Grants/Research Support: CSL Behring, Dyax, Pharming, Shire HGT, Viropharma Pharmaceuticals. Speaker's Bureau: CSL Behring, Dyax, Shire HGT, and Viropharma Pharmaceuticals. M. Magerl is or has been a speaker/consultant for Jerini, Shire, Sobi, Viropharma. I. Boccon-Gibod: CSL Behring, Shire, and Viropharma. B. Ritchie (research): CSL Behring, Dyax, Pharming, and Shire. B. L. Zuraw is a consultant of Dyax, Shire, BioCryst; research support: Shire. M. Maurer is or has been a speaker/consultant for and/or has received research funding from Biocryst, Jerini, Shire, and Viropharma. I. Martinez-Saguer (research): CSL Behring, Shire, Viropharma, and Pharming and speaker fees from CSL Behring and Shire. Advisory Boards for CSL Behring, Shire, and Pharming.

## Competing interests

The authors declare that they have no competing interests.
